# Therapeutic effects of flurbiprofen axetil on mesenteric traction syndrome: randomized clinical trial

**DOI:** 10.1186/s12893-017-0286-y

**Published:** 2017-08-11

**Authors:** Hidemasa Takahashi, Dai Shida, Kyoko Tagawa, Ryo Iwamoto, Makoto Arita, Hiroyuki Arai, Takeo Suzuki

**Affiliations:** 1 Department of Anesthesiology, Tokyo Metropolitan Bokutoh Hospital, 4-23-15, Koto-bashi, Sumida-ku, Tokyo, 130-8575 Japan; 2 Colorectal Surgery Division, National Cancer Center Hospital, 5-1-1 Tsukiji, Chuo-ku, Tokyo, 104-0045 Japan; 3 Business-Academia Collaborative Laboratory, Graduate School of Pharmaceutical Sciences, University of Tokyo, 37-3-1, Hongo, Bunkyo-ku, Tokyo, 113-0033 Japan; 4 Department of Health Chemistry, Graduate School of Pharmaceutical Sciences, The University of Tokyo, Tokyo, Japan

**Keywords:** Mesenteric traction syndrome, Flurbiprofen axetil, Prostacyclin, 6-keto-prostaglandin F1α, Colorectal cancer

## Abstract

**Background:**

This study aimed to reveal the appropriate timing for the intravenous administration of flurbiprofen axetil for preventing mesenteric traction syndrome (MTS), caused by prostacyclin release.

**Methods:**

In this prospective, randomized, clinical study, forty-five patients who were undergoing elective surgery for colorectal cancer via laparotomy were enrolled. Patients were randomly divided into 3 groups: a preoperative group (*n* = 16) receiving flurbiprofen axetil directly before surgery; a post-MTS group (*n* = 14) receiving following MTS onset; and a control group (*n* = 15) who were not administered flurbiprofen axetil. 6-keto-PGF1α, a stable metabolite of prostacyclin, levels were measured and mean blood pressures were recorded.

**Results:**

In the preoperative group, 6-keto-PGF1α levels did not increase, blood pressure levels did not decrease, and no facial flushing was observed. In both the post-MTS and control groups, 6-keto-PGF1α levels increased markedly after mesenteric traction and blood pressure decreased significantly. The post-MTS group exhibited a faster decreasing trend in 6-keto-PGF1α levels and quick restore of the mean blood pressure, and the use of vasopressors and phenylephrine were lower than that in the control group.

**Conclusions:**

Even therapeutic administration of flurbiprofen axetil after the onset of MTS has also effects on MTS by suppressing prostacyclin production.

**Trial registration:**

Clinical trial number: UMIN000009111. (Registered 14 October 2012)

## Background

Hemodynamic changes during surgery are a significant risk factor for postoperative complications and prolonged postoperative hospital stay [1, 2]. Intraoperative bleeding and sympathetic blocks in anesthesia are among the causes of hemodynamic changes during surgery. In addition, they can also be caused by the surgical procedure itself, such as that involving traction of the mesentery. Hemodynamic changes due to mesenteric traction were reported by Seltzer et al. in 1985 [3]. Mesenteric traction leads to increased shear stress on endothelial cells of mesenteric vessels and subsequent liberation of prostacyclin (PGI2) [4–7].

Hemodynamic changes occurring due to mesenteric traction are characterized by sudden tachycardia, hypotension, and facial flushing. This phenomenon is called mesenteric traction syndrome (MTS) [6]. We recently analyzed the circulatory dynamics of MTS by the Flo Trac sensor and demonstrated that systemic vascular resistance index (SVRI) fell down during MTS by 15.1%, indicating the close relationship between MTS and SVRI [8]. Because MTS is caused by the synthesis and secretion of prostacyclin, its onset can be confirmed by a rise in 6-keto-prostaglandin F1α (6-keto-PGF1α) levels, which is a stable end-product of prostacyclin metabolis [4, 5]. Thus, whereas the definitive diagnosis of MTS requires the measurement of the level of 6-keto-PGF1α, the clinical diagnosis is based on the occurrence of facial flushing.

The administration of non-steroidal anti-inflammatory drugs (NSAIDs) that are intended to inhibit the synthesis of prostacyclin seems to be able to prevent the onset of MTS [4, 7, 9]. Injectable formulations of NSAIDs are easy to use for the anesthesiologist. Recently the preoperative administration of flurbiprofen axetil, an injectable NSAID formulation, is reported to be effective in preventing MTS [10, 11]. According to pharmaceutical attached document of flurbiprofen axetil, this drug requires only 6.7 min after injection to reach the maximum blood level [12]. Therefore, it may be possible to utilize this drug after the onset of MTS. However, it is unknown whether flurbiprofen axetil should be administered prior to the start of the surgery or immediately after the confirmation of the onset of MTS as therapeutic administration. Thus, it is of interest to determine the appropriate timing of administration of NSAIDs for MTS.

## Methods

### Inclusion criteria

Patients undergoing elective surgery for colorectal cancer at our hospital were enrolled in this prospective, randomized clinical trial. This study was approved by the institutional Review Board (IRB) of Tokyo Metropolitan Bokutoh Hospital (IRB code: 15-Heisei23). Written informed consent was obtained from all patients who participated. This study was registered in UMIN-CTR (UMIN000009111). Apart from this study, UMIN000009111 included the pilot study with two arms (preoperative group and control group), the results of which we already reported previously [8].

### Exclusion criteria

Patients aged <20 years, or >82 years, and those with renal dysfunction, hepatic failure, peptic ulcer, aspirin-induced asthma, or arrhythmia were excluded. Patients who were taking steroids or nonsteroidal antiinflammatory drugs including aspirin were also excluded from the study. In addition, the cases that began with not colorectal surgery but the other operation such as liver resection will be excluded from the study because in such cases surgeons usually do not pull mesentery.

### Sample size calculation

According to the previous MTS study [10], 6-keto-PGF1α levels at time 20 min of preoperative group were 1078.2 ± 784.1 pg/ml, and these of control group were 20.5 ± 5.2 pg/ml. As for these of postoperative group, there were no previous available data and thus we hypothesized the smallest clinically relevant difference in 6-keto-PGF1α levels as 500 ± 650 pg/ml in postoperative group, almost half of its of preoperative group, which we would like to detect in a statistical test. Employing a Bonferroni correction, calculation of the sample size with type I error rate of 0.56% (= 5%/9) and power level as 80%, sample size needs 41 cases. Thus, we set 45 cases (15 cases for each three group, just like the previous MTS study [10]), and we planned to recruit 45 patients in our study.

### Study protocol

Forty-five patients, who were scheduled to undergo elective surgery for colorectal cancer via laparotomy from October 2012 to January 2013 were enrolled. Using a random number table, patients were randomly divided into 3 groups as follows: a preoperative group receiving flurbiprofen axetil (Ropion; Kaken Pharmaceutical, Tokyo, Japan) directly before surgery, a post-MTS group receiving flurbiprofen axetil following MTS onset, and a control group who were not administered flurbiprofen axetil. Patients in the preoperative group (*n* = 16) were administered flurbiprofen axetil (1 mg/kg; maximum dose = 50 mg) intravenously after induction of anesthesia, just prior to the initial incision. Patients in the post-MTS group (*n* = 14) received intravenous flurbiprofen axetil (1 mg/kg; maximum dose = 50 mg) after MTS onset. In this study, MTS was defined as a flushing of the face during an abdominal surgical procedure within 1 h after the initiation of surgery, and was diagnosed by two anesthesiologists [13]. If we could not confirm MTS onset, flurbiprofen axetil was not administered. Patients in the control group (*n* = 15) were not administered flurbiprofen axetil.

When hypotension requiring treatment were observed, ephedrine (Ephedrine; Nichi-Iko Pharmaceutical, Toyama, Japan), phenylephrine (Neosinejin; Kowa Company, Nagoya, Japan), or fluid (crystalloid solution or colloid solution), or all three were used at the discretion of the anesthesiologist in charge.

Throughout the perioperative care, all the patients were treated by our enhanced recovery after surgery (ERAS) protocols [14, 15]. No pre- and postoperative fasting (provision of oral nutrition) as well as intensive pre-admission counselling, avoidance of sodium/fluid overload, intraoperative warm-air body heating, enforced postoperative mobilization, and multimodal team care were among the main changes brought about by the introduction of ERAS protocols to our hospital [14, 15]. The course of the surgical procedure included peritoneal incision, opening of the abdomen, liver palpation (abdominal examination), unfolding of the colon, resection, and anastomosis; the same surgical team performed all the operations.

The subjects in each study arm were assigned in blinded fashion to the surgeons; however, the study design remained single-blind for the study team’s anesthesiologist.

All patients in all groups received general anesthesia in combination with epidural anesthesia. After admission into the operating room, an epidural catheter was inserted. Anesthesia was induced with propofol (1.5–2 mg·kg^−1^), rocuronium (0.6 mg·kg^−1^), sevoflurane (1–3%), remifentanyl (0.25–0.5 μg·kg^−1^·min^−1^), and ephedrine and dopamine as needed. After intubation, anesthesia was maintained with oxygen, air, sevoflurane (1.2–1.4%), and remifentanyl (0.1–0.25 μg·kg^−1^·min^−1^). Then, the radial artery was cannulated for continuous measurement of arterial pulse pressure. Following administration of 0.375% ropivacaine (4–6 mL) through the epidural catheter, the surgery was started. The tidal volume setting of the mechanical ventilator was 8–10 mL/kg.

To confirm the synthesis of prostacyclin, which triggers MTS, we measured the levels of 6-keto-PGF1α, a stable metabolite of prostacyclin. Because a previous report described the mean onset time of MTS to be 16 ± 5 min after skin incision with symptoms persisting over approximately 30 min [16], blood was collected from an arterial line at the initiation of surgery (T0) as well as at 15, 30, and 60 min after the initiation of surgery (T15, T30, and T60, respectively). The samples were refrigerated and centrifugal separation was rapidly performed. Then, blood plasma was subjected to cryopreservation. 6-Keto-PGF1α levels within the samples were measured using enzyme-linked immunosorbent assay (Enzo Life Sciences, Inc.) at the Department of Health Chemistry, Graduate School of Pharmaceutical Sciences, University of Tokyo.

### Statistical analyses

Results were displayed as mean value ± standard deviation (mean ± SD), and statistical processing was performed as follows. The statistical methods used for comparison between the groups were Chi-squared test for frequency data. Comparison of frequency data between 2 groups was performed using the independent *t*-test. As for 6-keto-PGF1α levels, an analysis of covariance (ANCOVA) comparing each pair out of the three intervention groups were used for analyses at each of time points (e.g. T15, T30, T60 for 6-keto-PGFIα level), each time taking the baseline measurement (e.g. 6-keto-PGFIα level at T0) as a covariate (both 6-keto-PGF1α levels and mean blood pressure). Since multiple tests were performed, a correction for multiple testing, a Bonferroni, was employed (In these cases, *P* < 0.0056 (0.05/3 × 3) was considered statistically significant). As for mean blood pressure, the Student’s t-test was used for comparing two time points (e.g. T0 as the baseline measurement, and T15) in each intervention group, and since multiple tests were performed, a correction for multiple testing, a Bonferroni, was employed (In these cases, *P* < 0.017 (0.05/3) was considered statistically significant). Because mean blood pressure was greatly influenced by the use of vasopressors and phenylephrine with various strength, analyses of its changes at T30, T45, and T60 were not performed, which were beyond our concern. Statistical analysis was performed using Dr. SPSS II for Windows (Ver. 11), and *P* < 0.05 was considered statistically significant unless otherwise mentioned.

## Results

### Patients

**Fig. 1:**
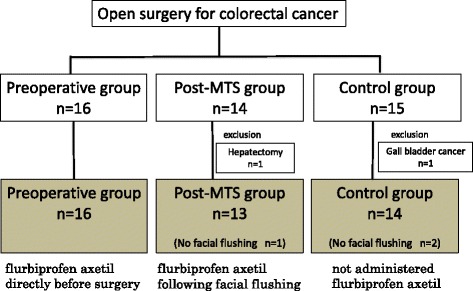
Flowchart depicting the study population. Flurbiprofen axetil was administered to 16 patients in the preoperative group and 12 patients who experienced facial flushing in the post-MTS group. Flurbiprofen axetil was not administered to one patient in the post-MTS group and 14 patients in the control group

There were 16 patients in the preoperative group, 14 in the post-MTS group, and 15 in the control group (Fig. 1). One patient from the post-MTS group was excluded from the study because the surgery began with liver resection rather than lower GI tract surgery because of detection of liver metastasis upon liver palpation. One patient from the control group was excluded from the study because of colon metastasis by gallbladder carcinoma. Thus, finally 16 patients in the preoperative group, 13 in the post-MTS group, and 14 in the control group were investigated. Facial flushing was observed in 12 patients (92%) in the post-MTS group and in 12 patients (86%) in the control group. These three patients, one patient in post-MTS group and two patients in control group who did not show facial flush, were also included in the analysis. Patient characteristics and basic operative data are presented in Table 1 (Fig. 1).

**Table 1:** Comparison of anthropometric characteristics and operative date between the preoperative group, the post-MTS group, and the control group

	Preoperative group	Post-MTS group	Control group	*P* value
Number of cases	16	13	14	
Gender ratio (male:female)	13:03	8:5	9:5	0.49
Age (y)	67.3 ± 11.3	69.0 ± 8.2	69.1 ± 10.2	0.85
Height (cm)	160.9 ± 9.7	162.6 ± 8.8	157.5 ± 12.3	0.43
Weight (kg)	59.1 ± 9.3	58.0 ± 13.3	55.8 ± 15.6	0.77
Body surface area (m2)	1.62 ± 0.16	1.61 ± 0.2	1.52 ± 0.22	0.35
Use of epidural anesthesia	16 (100%)	13 (100%)	14 (100%)	1
Use of dopamine	14 (87.5%)	12 (92.3%)	13 (92.9%)	1
Use of vasopressors (times)	0.8 ± 1.0	2.3 ± 1.2	6.1 ± 3.6	<0.05
Use of ephedrine (mg)	2.2 ± 3.1	1.2 ± 2.2	3.9 ± 4.9	0.14
Use of Phenylephrine (mg)	0.03 ± 0.08	0.24 ± 0.19	0.58 ± 0.45	<0.05
Operation time (min)	176 ± 44	223 ± 69	238 ± 99	0.06
Amount of bleeding (ml)	450 ± 436	826 ± 1013	802 ± 804	0.33
Amount of fluid (ml)	1693 ± 724	2975 ± 2336	2301 ± 1130	0.09

*n.s.* not significant

### 6-keto-PGF1α levels

**Fig. 2:**
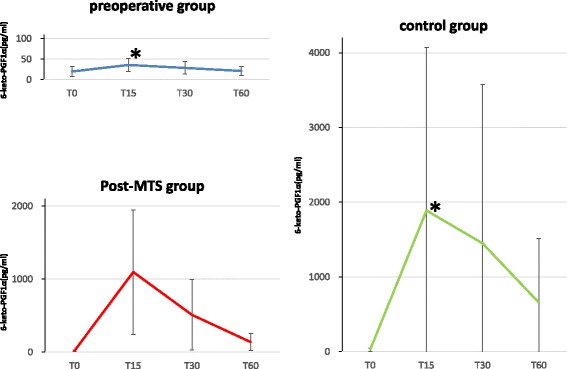
Trend for changes in mean 6-keto-PGF1α. T0 is the time point at the initiation of surgery. T15, T30, and T60 are at 15, 30, and 60 min after the initiation of surgery, respectively. Compared to the preoperative group, statistically significant increase of 6-keto-PGF1α levels were observed in the control group at T15. *,*P* < 0.0056, compared each pair out of the three intervention groups by ANCOVA combined with a Bonferroni correction

The changes in 6-keto-PGF1α levels occurring in the first 60 min of surgery are shown in Fig. 2. Compared to the preoperative group (36 ± 16 pg/ml), statistically significant increase of 6-keto-PGF1α levels were observed in the control group at 15 min after the initial incision (T15) (1889 ± 2179 pg/ml; *P* = 0.002), whereas increase of 6-keto-PGF1α levels in the post-MTS group was statistically not significant (1097 ± 853 pg/ml; *P* = 0.06). As shown in Fig. 2, the post-MTS group (*n* = 12) exhibited a faster decreasing trend in 6-keto-PGF1α levels as compared to the control group (*n* = 12) (at T30; 510 ± 483 pg/ml in post-MTS group, 1448 ± 2130 pg/ml in control group). At T30, the differences of 6-keto-PGF1α levels among three groups were not statistically significant (pre vs control; *P* = 0.0168, post vs control; *P* = 0.231, pre vs post; *P* = 0.516). Similarly, at T60, the differences of 6-keto-PGF1α levels among three groups were not statistically significant (pre vs control; *P* = 0.0057, post vs control; *P* = 0.043, pre vs post; *P* = 0.7987).

### Mean blood pressure soon after the initiation of surgery

**Fig. 3:**
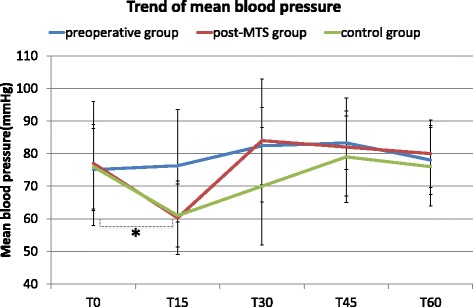
Trend for changes in mean blood pressure. T0 is the time point at the initiation of surgery. T15, T30, and T60 are at 15, 30, and 60 min after the initiation of surgery, respectively. At T15, in the preoperative group and in the control group, mean blood pressure significantly decreased compared to these at T0. *,*P* < 0.017 (0.05/3), t-test with a Bonferroni correction

Figure 3 shows the changes in mean blood pressure soon after the initiation of surgery. Mean blood pressure decreased significantly during the first 15 min of surgery (T0–T15) in the post-MTS and control groups as compared with the preoperative group (in the post-MTS group, from 77 ± 19 mmHg to 60 ± 11 mmHg (*P* = 0.004); in the control group, from 76 ± 13 mmHg to 61 ± 10 mmHg (*P* = 0.001); in preoperative group, from 75 ± 13 mmHg to 76 ± 17 mmHg (*P* = 0.58)). The use of vasopressors and phenylephrine were lower than that in the control group (the post-MTS group vs. the control group: 2.3 ± 1.2 vs 6.4 ± 3.7 times, 0.24 ± 0.2 vs 0.65 ± 0.44 mg, respectively; *P* < 0.05) (Table1).

### Facial flushing versus no facial flushing

In the post-MTS and control groups wherein the patients did not receive prophylactic flurbiprofen axetil, no facial flushing was observed in 3 patients (1 patient in post-MTS group and 2 patients in the control group). Their characteristics were compared with the remaining patients (12 patients in the post-MTS group and 12 patients in the control group) who experienced facial flushing. Irrespective of facial flushing, the 6-keto-PGF1α levels increased markedly during the first 15 min of surgery (T0–T15) in all patients (without facial flushing, from 16.4 ± 14.1 pg/mL to 602.6 ± 324.4 pg/mL; with facial flushing, from 24.9 ± 19.4 pg/mL to 1620.7 ± 1766.0 pg/mL). In addition, the mean blood pressure decreased over the same time period (T0–T15) (with facial flushing, from 76 ± 15 mmHg to 60 ± 10 mmHg; without facial flushing, from 78 ± 29 mmHg to 64 ± 13 mmHg).

### The length of hospital stay after surgery

Among three groups, there was no differences in the length of hospital stay after surgery (8.0 ± 4.2 days in the preoperative group, 11.5 ± 11.0 days in the post-MTS group, and 14.0 ± 13.8 days in the control group). And, between facial flushing patients and no facial flushing patients, there were no statistically significant differences between the two groups with respect to the length of hospital stay after surgery (with facial flushing, 12.9 ± 13.0 days; without facial flushing, 12.0 ± 7.8 days).

## Discussion

In this study, we demonstrated not only the prophylactic effect but the therapeutic effect of flurbiprofen axetil on MTS. We revealed that flurbiprofen axetil suppresses prostacyclin formation through measurements of its metabolites and that without the prophylactic administration of this COX inhibitor prostacyclin formation increased regardless of the presence or absence of facial flushing. The synthesis and secretion of prostacyclin was quickly inhibited in the post-MTS group following the administration of flurbiprofen axetil, and 6-keto-PGF1α levels were decreased faster in this group than in the control group. As a result, the time required to recover from the decreased blood pressure caused by MTS was shortened, and lower dosages of vasopressors and phenylephrine were needed. Thus, hypotension after MTS onset, facial flushing, had better be treated by not only alpha agents but also flurbiprofen axetil, or other NSAIDs that blocks the formation of prostacyclin, if there is no contraindication. These results were acceptable because flurbiprofen axetil, in contrast to other internal or suppository NSAIDs, is an injectable formulation and requires only several minutes to reach the maximum blood level [12, 17, 18].

The preoperative administration of flurbiprofen axetil inhibited the synthesis and secretion of prostacyclin, and could effectively prevent hypotension immediately after the initiation of surgery. These results were compatible with previous reports [10, 11]. In contrast, we revealed that without the prophylactic administration of flurbiprofen axetil, the levels of 6-keto-PGF1α increased regardless of the presence or absence of facial flushing.

Although the definitive diagnosis of MTS is based on an increase in 6-keto-PGF1α levels, MTS onset are clinically diagnosed based on facial flushing as well as sudden tachycardia and hypotension. In patients who did not receive flurbiprofen axetil preoperatively, the 6-keto-PGF1α levels increased regardless of the presence or absence of facial flushing. In general, dark skinned individuals or those with significant anemia, or even a color-blind anesthesiologist may inhibit the recognition of facial flushing. These results indicate that facial flushing does not necessarily occur at the onset of MTS, suggesting the need for a re-examination of the clinical diagnostic criteria of MTS including hypotension, tachycardia and facial flushing.

As described above, this study was the first to administer flurbiprofen axetil after MTS onset and verify its effects. However, without the administration of flurbiprofen axetil, the mean blood pressure was reduced by approximately 19.7% on average between the T0 and T15. In an observational study involving 1064 adult cases of non-cardiac surgery, Monk et al. [2] reported that when the intraoperative systolic blood pressure dropped <80 mmHg, the mortality rate increased 1.036-fold each minute. Tassoudis et al. [1] conducted a study targeting 100 adult cases of abdominal surgery for which surgery of over 2 h was planned and reported that intraoperative hypotension (defined as mean blood pressure of <60 mmHg, or mean blood pressure of <70 mmHg decreased by 30% or more from the baseline) continued for ≥10 min, postoperative hospital days increased and the incidence of complications increased. Thus, intraoperative hypotension is a risk for the onset of perioperative complications and increased postoperative days in the hospital; therefore, prophylactic administration of flurbiprofen axetil before surgery is a reasonable therapeutic choice to prevent MTS and its subsequent consequences, especially, in patients who cannot increase cardiac output to compensate for reduced vascular resistance, which would be those with some forms of heart failure or cardiac valvular disease. A warning is necessary in administering COX inhibitor to the patients with renal dysfunction because of the potential renal effect of this agent.

In this study, the length of postoperative hospital stay for each group was also examined. Thus far, no studies have evaluated the prognosis of MTS. In patients who received prophylactic administration of flurbiprofen axetil, the length of postoperative hospital stay was shorter than in those who were not administered prophylactic flurbiprofen axetil; however, no statistically significant difference was noted. This was possibly due to the small sample size, which was inadequate for assessing perioperative outcomes.

## Conclusions

This study was the first to administer flurbiprofen axetil after MTS onset and verify its effects such as a faster decreasing trend in 6-keto-PGF1α levels and restoring the mean blood pressure as soon as possible. Whereas preoperative prophylactic administration of flurbiprofen axetil could completely suppress and prevent the onset of MTS, even therapeutic administration of flurbiprofen axetil after the onset of MTS has also some effects on MTS by suppressing prostacyclin production.

## Abbreviations

6-keto-PGF1α: 6-keto-prostaglandin F1α
ERAS: Enhanced recovery after surgery
MTS: Mesenteric traction syndrome
NSAIDs: Non-steroidal anti-inflammatory drugs
